# Identification of m6A-associated genes as prognostic and immune-associated biomarkers in Wilms tumor

**DOI:** 10.1007/s12672-023-00817-w

**Published:** 2023-11-08

**Authors:** Yingquan Zhuo, Wengqi Zhang, Jun Du, Hua Jiang, Guangtang Chen, Xiaoyun Feng, Huajian Gu

**Affiliations:** 1 Department of Pediatric Surgery, The Affiliated Hospital of Guizhou Medical University, Guiyang, 550004 China; 2 School of Clinical Medicine, Guizhou Medical University, Guiyang, 550004 China; 3 Department of Anesthesiology, The Affiliated Hospital of Guizhou Medical University, Guiyang, 550004 China; 4 School of Basic Medicine, Guizhou Medical University, Guiyang, 550004 China

**Keywords:** Wilms tumor, m6A RNA methylation, Target genes, Immune microenvironment, Bioinformatics analysis

## Abstract

**Objectives:**

Wilms tumor (WT) is a common renal malignant tumor in children. We aimed to investigate the potential prognostic value of m6A-related genes and their relationship to the immune microenvironment in WT.

**Methods:**

RNA-seq data and clinical information from 121 WT and 6 normal samples were obtained from the University of California Santa Cruz Xena database. We used various bioinformatics analysis tools to analyze these data and verify the expression level of m6A-related genes by experiments.

**Results:**

Four m6A-related genes were successfully screened, including ADGRG2, CPD, CTHRC1, and LRTM2. Kaplan–Meier survival curves showed that the four genes were closely related to the prognosis of WT, which was also confirmed by receiver operator characteristic curves. Subsequently, in the immune microenvironment of WT, we discovered that Th1_cells were positively correlated with ADGRG2, CCR was negatively correlated with CPD, CCR was positively correlated with CTHRC1, APC_co_stimulation, CCR, Macrophages, inflammation-promoting cells, Treg, and Type_II_IFN_Reponse were negatively correlated with LRTM2. Finally, qRT-PCR showed that expression levels of the four genes were upregulated in the nephroblastoma cell lines (G-401, SK-NEP-1, and WT-CLS1) compared with the human embryonic kidney cell lines (293T).

**Conclusions:**

Taken together, our study first time screened the m6A-related genes and revealed that ADGRG2, CPD, CTHRC1, and LRTM2 are the prognostic and immune-associated biomarkers in WT.

## Introduction

Wilms tumor (WT) is the most common kidney tumor in children, affecting one in 10,000 children, accounting for about 90% of all pediatric kidney tumors and approximately 5% of all childhood cancers [1]. In the United States, the median age of onset of WT is 38 months, and the main clinical symptom is an asymptomatic abdominal mass [2, 3]. According to Children’s Oncology Group (COG) staging systems, WT can be divided into stages I–IV from low to high based on the degree of tumor invasion, and stage V refers to the rare bilateral WT [4]. Although the WT survival rate increased from 30 to 80% with surgery in addition to radiotherapy and chemotherapy, the child has to endure this long, multimodal, and toxic regimen [5]. Therefore, it is still necessary to find better treatments. Understanding the pathogenesis of WT is conducive to developing new therapies, but the pathogenesis of WT is still not fully understood. Alfred Knudson and colleagues conducted a statistical analysis in 1972 on familial/bilateral and sporadic/unilateral cases of Wilms’ tumor (WT) to elucidate the genetic basis of bilateral and unilateral retinoblastoma. With the discovery of the first WT predisposition gene, WT1, Knudson’s theory regarding WT was substantiated in 1990 [6]. WT1, locus at chromosome 11p13, is inactivated in 15% of sporadic WT, and constitutive activation of β-catenin in the Wnt pathway associated with kidney differentiation has been observed in these tumors. In addition to the WT1 locus at chromosome 11p13 and the telomeric imprinting locus at 11p15, loss of heterozygosity (LOH) has also been confirmed at chromosomes 1p, 16q, and 7p in sporadic WT, suggesting the presence of unidentified tumor suppressor genes [7]. Mutations of WT1, CTNNB1, SIX1, SIX2, FGFR1, CHD4, and other genes have been reported in WT [8]. About 5% of WT patients have an underlying susceptibility gene syndrome; more than 50 such syndromes have been described [9]. Although some WT susceptibility genes have been identified, there is strong evidence that many more may exist [10]. Therefore, the study of WT-related genes is of great value in determining biomarkers of WT and discovering new therapeutic targets, which is conducive to developing personalized therapy.

In recent years, epigenetic inheritance has been studied intensively in WT [11]. Epigenetics means that the DNA sequence does not change, but the gene expression has changed heritably [12]. RNA modification is a crucial process in epigenetic regulation of post-transcriptional gene expression, the most common of which is RNA methylation. Methylation of the N6 position of adenine, called N6-methyladenine (m6A), is the most common internal modification of eukaryotic mRNAs [13]. M6A can be installed and removed by specific enzymes, classified as “writer”, “eraser,” and “reader” [14]. In general, abnormal expression of m6A disrupts the normal RNA modification process, directly interfering with mRNA processing, transport, translation, and degradation, leading to tumorigenesis and progression [15]. In childhood cancer research, the overexpression of m6A RNA methylation has been found in glioma, hepatoblastoma, nephroblastoma, neuroblastoma, osteosarcoma, medulloblastoma, retinoblastoma and acute lymphoblastic leukemia [16]. The target of m6A modification may be the key to promoting pediatric cancer treatment. Five m6A regulatory genes were found to be new therapeutic targets for neuroblastoma [17]. Utilizing m6AScores constructed through machine-learning strategies, two distinct m6A modification patterns have been uncovered in glioma, and the m6AScores are capable of predicting the molecular subtypes of low-grade gliomas, the abundance of immune infiltration, enrichment of signaling pathways, gene mutations, and prognosis [18]. m6A-associated genes exhibit significant appearance in hepatoblastoma, demonstrating potential in predicting disease course and therapeutic response [19]. A pan-cancer analysis study revealed a significant correlation between m6A RNA methylation and clinical outcomes, neoantigen burden, immune infiltration, and stemness [18]. Therefore, paying attention to the overexpression of m6A RNA methylation and m6A-related genes in tumor tissues may help to treat tumors further.

The occurrence of tumors is associated with immune surveillance disability [20]. Immunosuppressive microenvironment seems to be prevalent in WT. An animal study found that anti-tumor executive lymphocytes (such as CD4+ T cells, CD8+ T cells, NK cells, and NKT cells) in the WT high-risk group responded in an immunosuppressed state compared to the low-risk subgroup [21]. Studies have shown that patients with nephroblastoma with high CD8+ T lymphocyte infiltration have better clinical prognoses [22]. These findings not only imply the presence of an immunosuppressive tumor microenvironment in WT, but importantly, they highlight the prognostic significance of immune cell infiltration and immune dysregulation in determining clinical outcomes for WT patients. It seems feasible to treat tumors by activating the immune activity of the WT microenvironment. However, immunotherapy for most solid cancers in children has not shown an objective anti-tumor effect [23]. Lack of knowledge of the immune infiltration landscape of these tumors may be one of the reasons for the failure of immunotherapy.

Our study aimed to investigate the potential prognostic value of m6A-related genes and their relationship to the immune microenvironment in WT. This study obtained RNA-seq data and clinical information from 121 WT samples and 6 normal samples from the UCSC Xena website. We analyzed the relationship between m6A RNA methylation and the occurrence and development of WT and screened m6A RNA methylation-related genes. Based on these genes, WT clinical correlation analysis, immune infiltration correlation analysis, TF-miRNA-mRNA network construction, and targeted drug prediction were performed. Finally, Real-time quantitative PCR (qRT-PCR) was used to verify the expression of m6A-related genes in different WT cell lines. This study will help to deepen the understanding of the relationship between m6A and nephroblastoma and provide some evidence for the future detection of the role of m6A RNA methylation in nephroblastoma.

## Materials and methods

### Data collection

The RNA-seq data and clinical data of the TARGET-WT cohort were extracted from the University of California Santa Cruz (UCSC) Xena database (https://xenabrowser.net/datapages/), which included 121 WT patients and 6 normal samples. M6A RNA methylation regulators, including writers (METTL3, METTL14, RBM15, WTAP, ZC3H13, KIAA1429), readers (HNRNPC, YTHDC1, YTHDC2, YTHDF1, YTHDF2), and erasers (ALKBH5, FTO) were collected from previous research [24].

### GSVA analysis

M6A RNA methylation score was estimated using RNA-seq data of TARGET-WT patients through performing Gene Set Variation Analysis (GSVA) analysis, and the correlation between different clinicopathological features (Stage, Race, Gender, Histologic_Classification) and m6A score was analyzed. GSVA analysis was performed using the GSVA R package to estimate enrichment scores for the m6A gene sets in each WT sample. GSVA transformed gene expression data into an enrichment profile over the given gene sets, allowing the evaluation of pathway activity variation between samples. First, a kernel estimation of the cumulative density function was calculated for each gene’s expression across all samples. Then, a gene-set enrichment score was computed for each sample based on its rank in the distribution. This non-parametric approach did not require class label information and estimates variation of gene set enrichment over the samples independently of any specific phenotype. We applied GSVA using the m6A gene sets as input to obtain a sample-wise m6A enrichment score for the WT cohort.

### Identification of the module with significantly associated m6A RNA methylation score based on weighted gene co-expression network analysis (WGCNA) analysis

Based on the median value of all gene expression matrices, the 121 WT samples were divided into high and low-score groups of m6A RNA methylation, and the two groups were used as trait data to construct the co-expression network using WGCNA software. The samples were initially clustered to check the overall correlation of all the samples. Pearson’s correlation matrices were calculated for all gene expression data, and samples with low connectivity were removed as outliers to ensure the accuracy of downstream analysis. Next, we determined the soft thresholding power based on the scale-free topology criterion to obtain a scale-free gene co-expression network. The power value of β = 4 was chosen, resulting in an approximate scale-free topology index R^2^ of 0.86. The adjacency matrix was then constructed by raising the correlation matrix to the power β, representing the connection strengths between genes. A topological overlap matrix and dissimilarity measure were calculated using the adjacency matrix to estimate network interconnectedness. Modules were defined from the dendrogram based on the dynamic tree-cutting algorithm, which identified clusters of highly correlated genes. As recommended, we set the minimum module size to 300 genes to ensure medium-sized modules with biological significance. The cut height to merge modules was set at 0.25, resulting in 7 distinct modules. A threshold of 0.2 was used to filter the module eigengenes, retaining those that exhibited higher correlations for downstream analysis. Finally, module eigengenes were computed from the principal component analysis of each module, and the associations between modules and m6A methylation score were evaluated using Pearson’s correlation tests.

### Identification of differentially expressed genes (DEGs)

The DEGs between 121 WT patients and 6 control samples were screened using the limma (version 3.42.2) package in the R platform, and genes with |log_2_FC| > 0.5, *P* < 0.05 were regarded as DEGs. The DEGs were exhibited through volcano plots via the ggplot2 package (version 3.3.5). The heatmaps of DEGs were performed using the heatmap package (version 1.0.12). In addition, the DEGs between 61 high-score samples and 60 low-score samples of m6A RNA methylation were screened using the limma package (|log_2_FC| > 0.5, *P* < 0.05). The volcano plots and heat maps of DEGs were plotted using the R package. Moreover, the Venn diagram was constructed with the model genes significantly associated with m6A RNA methylation score, the DEGs between WT patients and control samples, and the DEGs between high and low score groups of m6A RNA methylation. The intersection genes were obtained as candidate hub genes for further analysis.

### Identification and analysis of hub genes

The 121 WT samples were divided into high and low-expression groups according to the median expression levels of candidate hub genes. The survival analysis was performed for both groups, and the hub genes were filtered. Subsequently, the diagnostic models were constructed using the hub genes. The receiver operator characteristic (ROC) curve calculated the area under the curve (AUC) area to investigate the effectiveness of the diagnostic models by the pROC (version 1.17.0.1). In addition, according to the median expression levels of hub genes, 121 WT samples were divided into high and low-expression groups, and all genes were analyzed by Gene set enrichment analysis (GSEA) analysis. Furthermore, the boxplot was used to display the expression level of the hub genes in different clinical features (Gender, Stage, Race, and Histologic_Classification).

### Immune cell infiltration landscape

The single sample gene set enrichment analysis (ssGSEA) algorithm evaluated the immune infiltration degrees based on 28 immune-related gene sets, including immune cell type, immune-related functions, and pathways. First, the GSVA package assessed the relative expression between 121 WT and 6 normal samples. Then, the heat map and box plots of immune cells were plotted. Moreover, the Spearman method calculated the correlation between hub genes and 28 immune gene sets. The heat map and correlation scatter figure were plotted using the ggplot2 package.

### Construction of transcription factor (TF)-mRNA-miRNA regulatory network

The TFs that regulate hub genes were retrieved using the NetworkAnalyst (https://www.networkanalyst.ca/) database. The hub genes were then predicted by the miRWalk (https://mirwalk.umm.uniheidelberg.de/) database. The parameters Score was set as 0.95, the Position was set as 3UTR, and miRDB was set as 1. Finally, the TF-mRNA-miRNA regulatory network was constructed by the Cytoscape package (version 3.6.1).

### Prediction of Potential Therapeutic Drugs

The Comparative Toxicogenomics Database (CTD) included precise data on interspecies chemical gene/protein interaction, the relationship between chemical and disease, and the relationship between genes and compounds. It contributed to understanding the molecular mechanisms underlying potentially variable susceptibility and environmental influences on conditions. The potential therapeutic drugs of hub genes were predicted using the CTD.

### Validation of the gene expression level by qRT-PCR

Four cultured cell lines with a density of 70–90% were divided into two groups, among which 293T was the human embryonic kidney cell group, and G-401, SK-NEP-1, and WT-CLS1 were the WT groups. The three Wilms tumor cell lines chosen for validation (G-401, SK-NEP-1, WT-CLS1) were selected to represent different histological subtypes relevant to the heterogeneity of the Wilms tumor. G-401 (product code: ATCC-CRL-1441) was originally derived from a malignant anaplastic nephroblastoma and exhibited epithelial morphology [25]. SK-NEP-1 (product code: ATCC-HTB-48) was established from a pediatric Wilms tumor patient and maintains mixed blastemal, epithelial, and stromal features [26]. WT-CLS1 (Cellosaurus accession: CVCL_5904) was developed from a lung metastasis of a relapse Wilms tumor case and demonstrated triphasic histology [27]. The 293T (product code: ATCC-CRL-3216) is an epithelial-like cell isolated from a patient’s kidney and was used as a normal control.

The qRT-PCR was used to verify the expression level of key genes. First, the total RNA was extracted using the Trizol reagent provided by Ambion company. Then, we used the SureScript-First-strand-cDNA-synthesis-kit First-Strand cDNA Synthesis Kit from the Servicebio company for reverse transcription reaction in the S1000™ Thermal Cycler PCR instrument. The qRT-PCR was performed using the 2xUniversal Blue SYBR Green qPCR Master Mix kit by CFX96 Connect real-time quantitative fluorescence PCR instrument. Primer sequences are shown in Table 1. GAPDH was used as an internal reference gene. The qRT-PCR reactions were as follows: 95 °C predenaturation for 1 min, and then 40 cycles of 95 °C denaturation for 20s, 55 °C annealing for 20s, and 72 °C extension for 30 s. The relative mRNA expression was also calculated using the 2^−△△Ct^ method. Eventually, GraphPad Prism 5 was used to plot the boxplots of expression levels and calculate the P values in the four cultured cell line groups.

**Table 1:** Quantitative real-time PCR primers of four hub genes

Gene name	Forward primer	Reverse primer
ADGRG2	GAGGCGGTATCTTTGTTGTGGA	CAGTGTGGTGGAGTTAGTGGAG
CPD	AGGATTGTGATTGTCCCTTCTC	TTCCACTGTCTGTACTGGCTTG
CTHRC1	TGCTGTCAGCGTTGGTATTTC	CATTTCAGGGCTTCCTTGGTC
LRTM2	CAGGTCGGGGATAACCCCT	CCCTCGGTAGGAGAACCACT
GAPDH	ACAACTTTGGTATCGTGGAAGG	GCCATCACGCCACAGTTTC

## Results

### The association between m6A RNA methylation with WT development

**Fig. 1:**
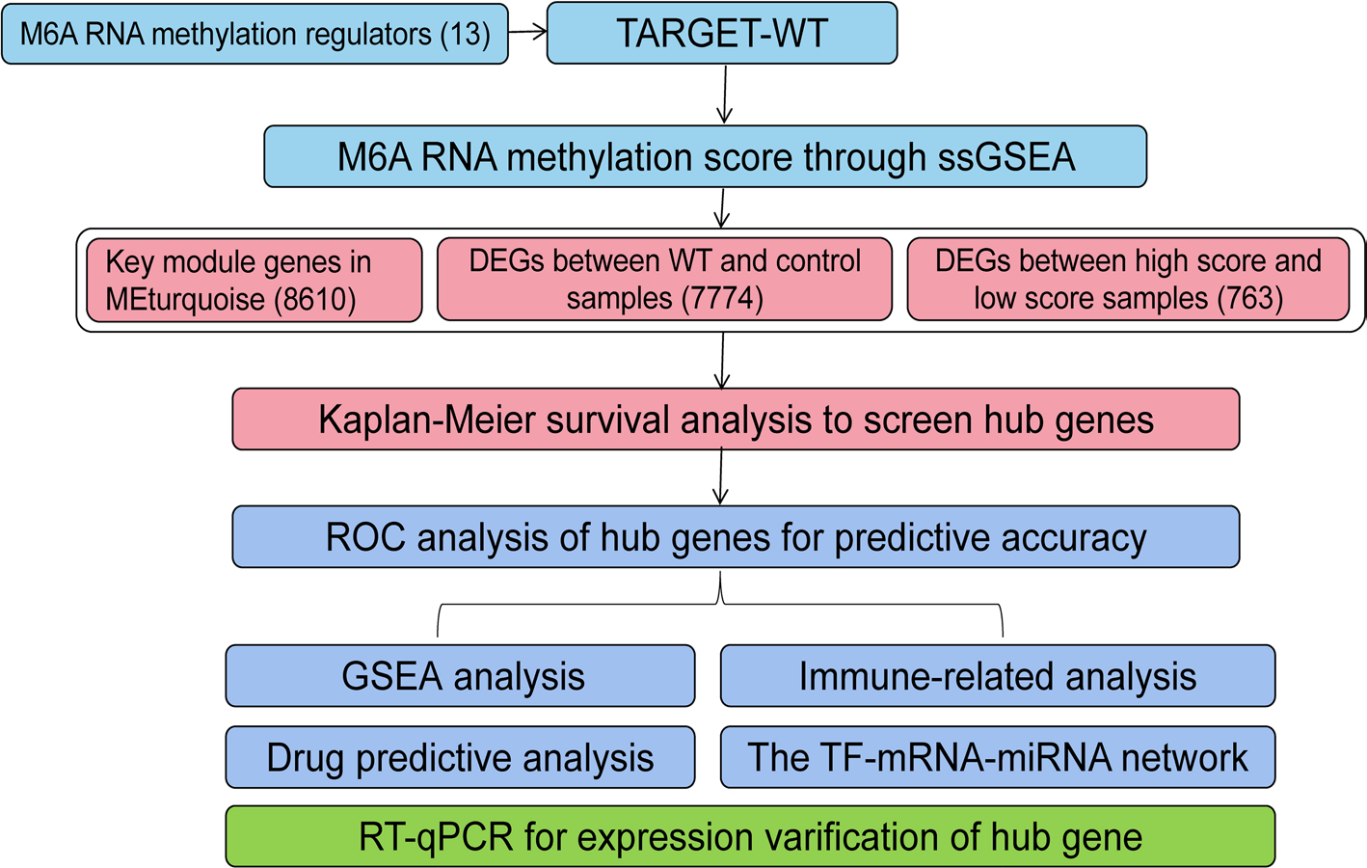
The workflow diagram of the current study

**Fig. 2:**
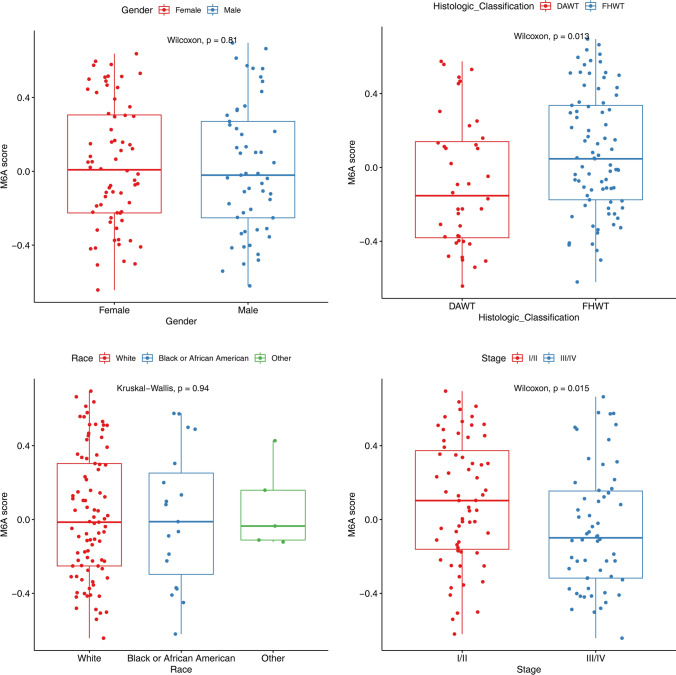
Relationship between m6A RNA methylation and different clinicopathological features. including Stage (Wilcoxon test, *P =* 0.015), Race (kruskal-wallis test, *P =* 0.94), Gender (Wilcoxon test, *P =* 0.81), Histologic_Classification (Wilcoxon test, *P =* 0.013)

The workflow diagram of the current study is exhibited in Fig. 1. The m6A RNA methylation score was calculated by GSVA package, and the correlation between different clinicopathological features (Stage, Race, Gender, Histologic_Classification) and m6A score was analyzed. The m6A RNA methylation score was significantly different at Stage and Histologic_Classification. Specifically, the m6A score was higher in Stage I/II and FHWT (Fig. 2).

### Identification of the module with significantly associated m6A RNA methylation score based on WGCNA analysis

**Fig. 3:**
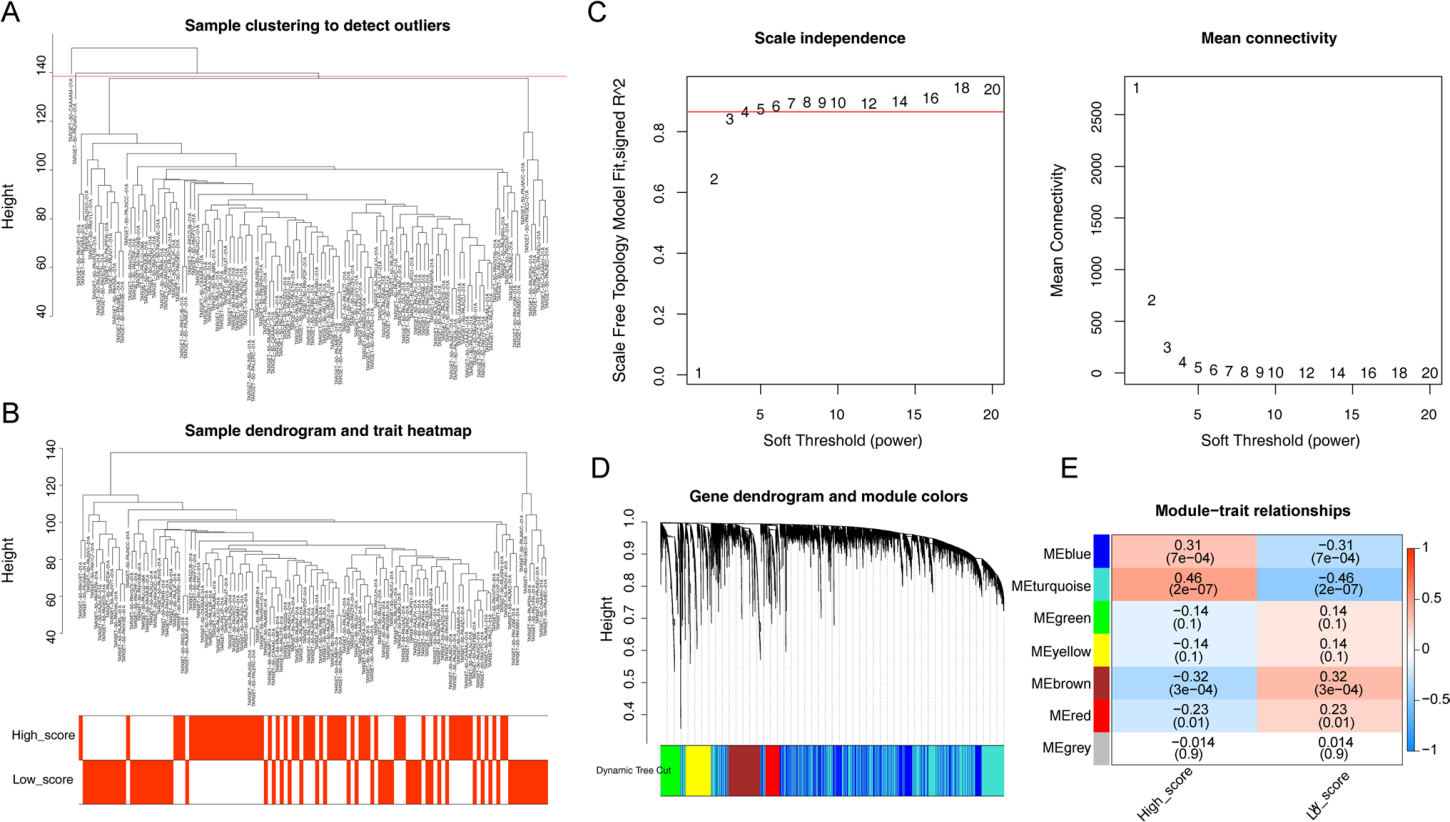
WGCNA for the key module genes that were significantly associated with m6A RNA methylation score. **A** Sample clustering to detect outliers. **B** Sample dendrogram and trait heatmap. **C** Analysis of the scale-free fit index and the mean connectivity for various soft-thresholding powers to determine the optimal soft-thresholding power, where 4 was considered as the most fit power value (R^2^ = 0.86). **D** The cluster dendrogram, according to gene expression that exhibits the co-expression modules with different colors. Each branch in the figure represents one gene. **E** Module-trait relationships heatmap to screen key module (MEturquoise, Cor = 0.46, *P =* 2e−07)

The WGCNA was used to identify the module genes with significantly associated m6A RNA methylation scores. Sample clustering results show that TARGET-50-CAAAAM-01 A and TARGET-50-PAJNAV-01 A were the abnormal outlier samples, and we eliminated this sample (Fig. 3A). The sample dendrogram and trait heatmap were exhibited in Fig. 3B. The soft threshold value = 4 was identified as the optimal soft threshold. When the ordinate scale-free fit index, signed R2 approached the threshold value of 0.86 (red line), the network was close to scale-free distribution, and mean connectivity was close to 0 (Fig. 3C). A total of 7 modules were obtained (Fig. 3D). Then the correlation between modules and high and low score groups of m6A RNA methylation was evaluated. The results showed that the MEturquoise module had the most significant correlation with the m6A RNA methylation score (Cor = 0.46, *P =* 2e−07) (Fig. 3E). Finally, we regarded MEturquoise as a key module with 8610 genes for subsequent analysis.

### Identification of DEGs

**Fig. 4:**
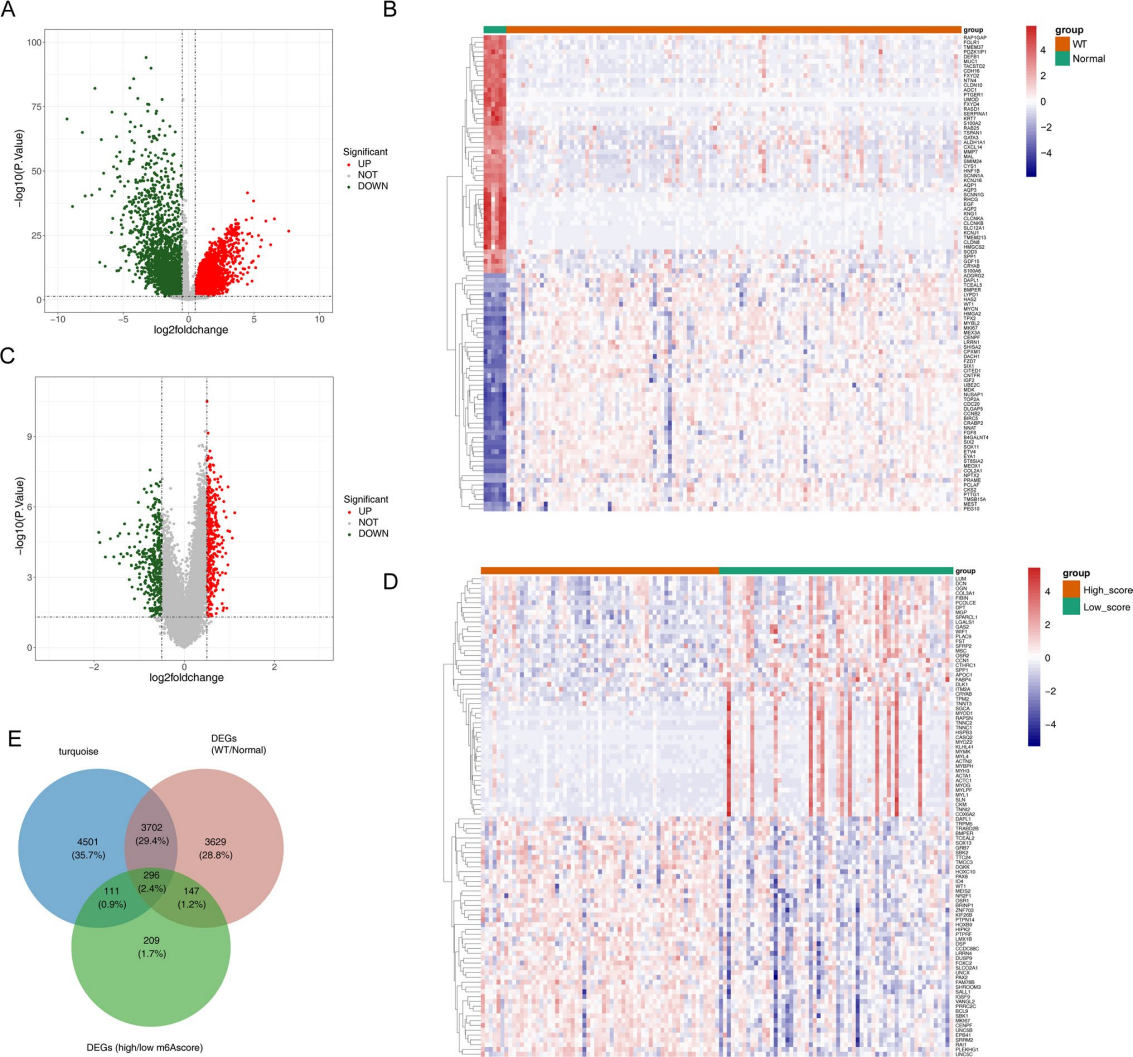
Identification of candidate hub genes. **A**, **B** Volcano and Heatmap of differentially expressed genes (DEGs) between WT patients and control samples with |log_2_FC| > 0.5, *P* < 0.05. **C** Volcano and **D** Heatmap of DEGs between high and low score samples with |log_2_FC| > 0.5, *P* < 0.05. **E** Venn diagram of 296 candidate hub genes by overlapping the key module genes, DEGs of WT and control samples, and DEGs of high and low score groups

In total, 7774 DEGs were retrieved between WT patients and normal samples, of which were 4338 upregulated genes and 3436 downregulated genes (Fig. 4A). The heat map of Top50 DEGs was shown (Fig. 4B). In addition, 763 DEGs were retrieved between a high score and low score sample that 399 genes were upregulated and 364 genes were downregulated (Fig. 4C). The heat map of Top50 DEGs was shown (Fig. 4D). Then 296 candidate hub genes were obtained by overlapping the 8610 MEturquoise module genes, 7774 DEGs of WT and control samples, and 763 DEGs of high and low score groups (Fig. 4E).

### Identification and analysis of hub genes

**Fig. 5:**
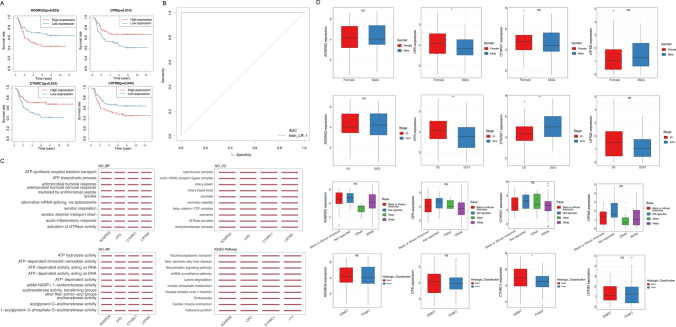
Identification of hub genes with excellent diagnostic significance for WT. **A** Kaplan–Meier survival curve of four DEGs_m6A with *P* < 0.05 that were identified as hub genes. **B** The receiver operator characteristic (ROC) curve for the four-hub genes-based diagnostic model. **C** Gene set enrichment analysis (GSEA) of four hub genes, including the mainly enriched gene ontology (GO) and Kyoto Encyclopedia of Genes and Genomes (KEGG) terms. **D** The hub gene expression levels in WT cohorts with different clinicopathological features (Stage, Race, Gender, Histologic_Classification) (Wiloxon test, *P* < 0.05)

To identify prognostic hub genes, the 121 WT samples were first divided into high and low-expression groups based on the median expression of each of the 296 candidate genes. Kaplan–Meier survival analysis was performed, and genes where expression levels were significantly associated with survival probability (p < 0.05) were retained (Fig. 5A). This yielded four genes that met the survival analysis criteria—ADGRG2, CPD, CTHRC1, and LRTM2. A logistic regression model was constructed using these four genes to evaluate their diagnostic utility. The model achieved an AUC of 1, indicating good diagnostic performance (Fig. 5B). Based on the median expression levels of the four hub genes, 121 WT patients were divided into two groups, and GSEA results indicated the four hub genes were enriched in ATP biosynthetic process, acute inflammatory response, cytochrome complex, alditol: NADP+ 1-oxidoreductase activity, Inositol phosphate metabolism pathway, etc. (Fig. 5C). Furthermore, the expression levels of CPD and CTHRC1 had significant differences in stage (Fig. 5D).

### Immune cell infiltration landscape

**Fig. 6:**
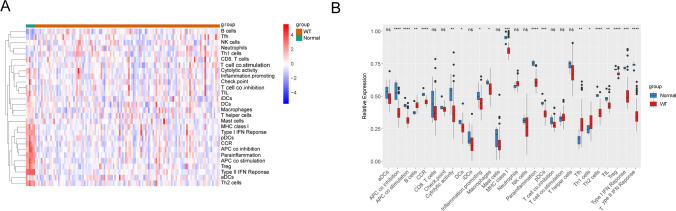
Immune cell infiltration in WT. **A**, **B** Heatmap and boxplot of 28 immune cells infiltration between the normal and WT groups (Wiloxon test, *P* < 0.05)

**Fig. 7:**
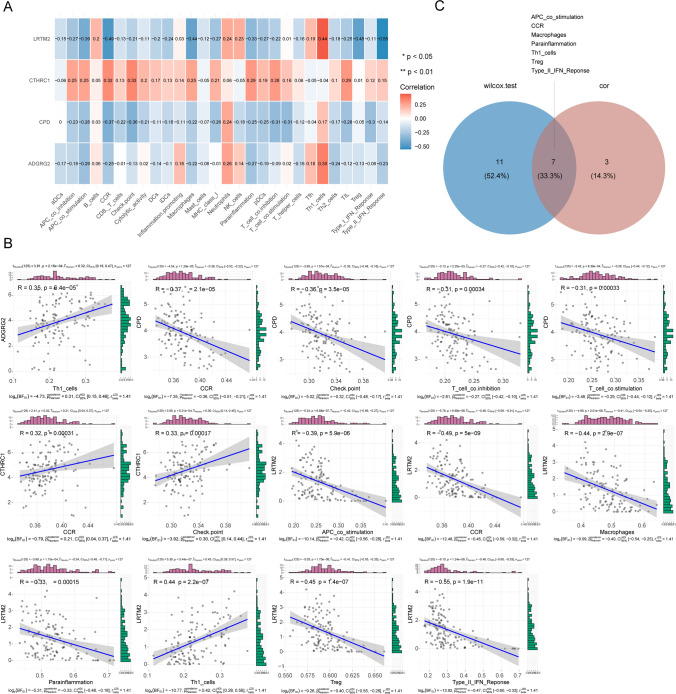
Relationship of hub gene and immune infiltration cells in WT. **A** Spearman correlation heatmap of hub genes and 28 immune cell types (*P* < 0.05). **B** Correlation scatter plot of hub genes and immune cell types with *P* < 0.05, |cor| > 0.3, Columnar diagrams at the top and right show gene expression and immune cell infiltration levels for each sample in the training set. **C** Venn diagram of key immune cell types by intersecting the differentially expressed immune cells and the immune cell types that were significantly correlated with hub genes with |cor| > 0.3

The immune cell infiltration analysis was performed, and the heat map exhibited the contents of 28 immune gene sets in the normal and WT groups (Fig. 6A). There were 18 immune gene sets (APC_co_inhibition, APC_co_stimulation, B_cells, CCR, Cytolytic_activity, DCs, Inflammation promoting cells, Macrophages, MHC_class_I, Parainflammation, pDCs, Tfh, Th1_cells, Th2_cells, TIL, Treg, Type_I_IFN_Reponse, and Type_II_IFN_Reponse) that showed a significant difference between normal and WT groups (P < 0.05) (Fig. 6B). Then the correlation coefficient and P value between hub genes and 28 immune gene sets were calculated (Fig. 7A). The 10 significantly correlated relationship of hub genes and immune genes were screened (P < 0.05, |cor| > 0.3). Finally, seven key immune gene sets (APC_co_stimulation, CCR, Macrophages, Parainflammation, Th1_cell, Treg, and Type_II_IFN_Reponse) were obtained by overlapping the 18 significant difference immune gene sets and 10 immune gene sets of |cor| > 0.3 (Fig. 7B). APC_co_stimulation, CCR, Macrophages, Parainflammation, Treg, and Type_II_IFN_Reponse were negatively correlated with LRTM2; Th1_cells were positively correlated with ADGRG2; CCR was negatively correlated with CPD; CCR was positively correlated with CTHRC1 (Fig. 7C).

### TF-mRNA-miRNA network

**Fig. 8:**
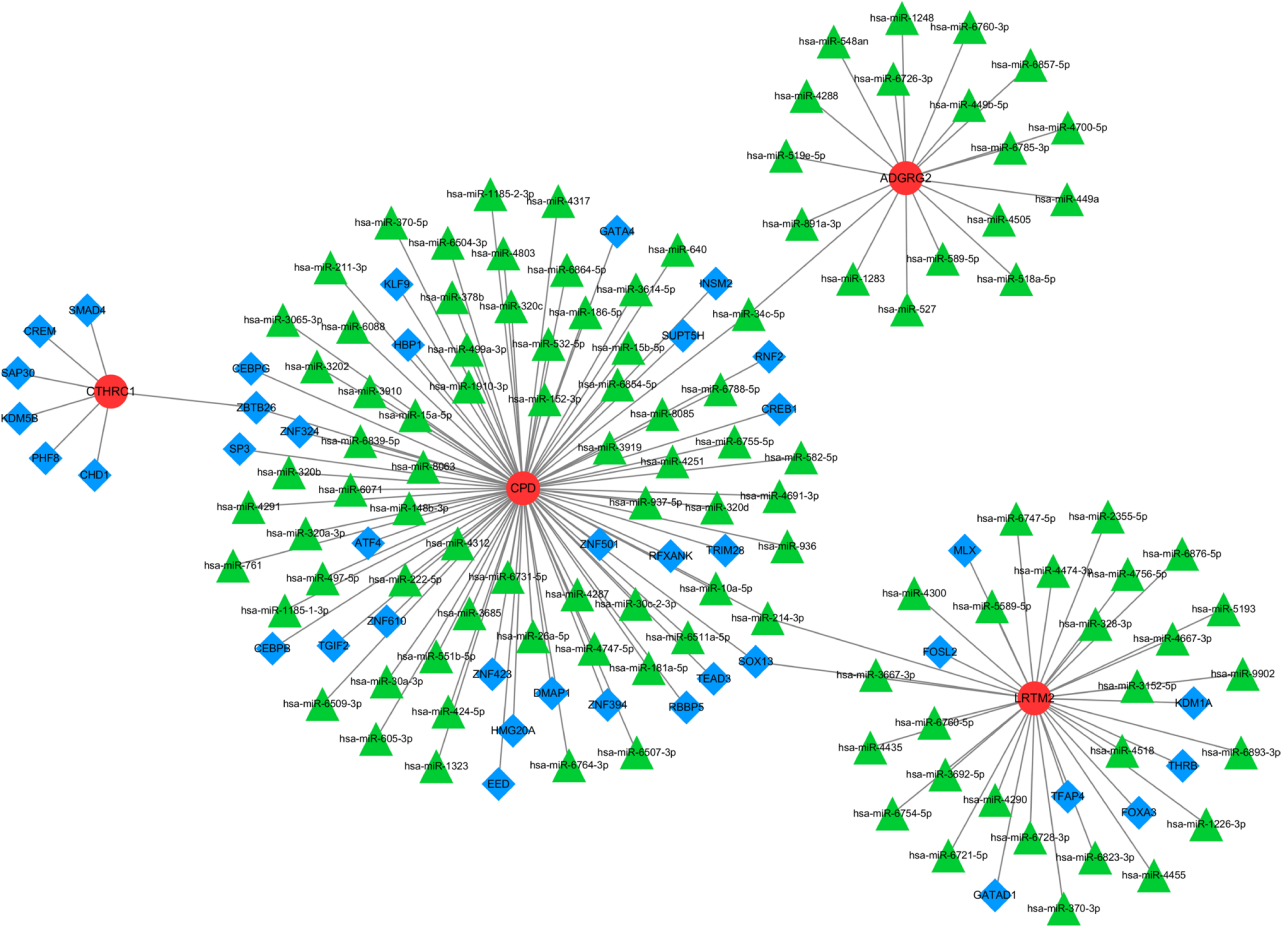
Construction of the TF-mRNA-miRNA network through NetworkAnalyst and miRWalk databases. Red circles represent Hub genes, green triangles represent miRNAs, and blue diamonds represent TFs

The TF-mRNA-miRNA regulatory network was constructed. NetworkAnalyst first predicted the 39 TFs that regulate hub genes. The miRWalk predicted 107 miRNA that targeted with hub genes. Then, the regulatory network was constructed by the 4 hub genes, 39 TFs, and 107 miRNA (Fig. 8). The network indicated CPD had the greatest number of interactions with TF and miRNA, and CPD was modulated by HMG20A, ZNF501, GATA4, hsa-miR-15b-5p, hsa-miR-370-5p, and hsa-miR-152-3p. CTHRC1 was only modulated by TFs, including KDM5B, SAP30, ZBTB26, etc. ADGRG2 was only modulated by hsa-miR-589-5p, hsa-miR-34c-5p, and hsa-miR-519e-5p.

### Identification of potential therapeutic drugs

The potential therapeutic drugs for WT were predicted by the four hub genes (Table 2). The results showed that ADGRG2 predicted 82 therapeutic drugs, including Benzo(a)pyrene, Aflatoxin B1, Tetrachlorodibenzodioxin, etc. The CPD predicted 91 therapeutic drugs, including bisphenol A, Testosterone, Tetrachlorodibenzodioxin, etc. The CTHRC1 predicted 81 therapeutic drugs, including Valproic Acid, 4-(5-benzo(1,3)dioxol-5-yl-4-pyridin-2-yl-1*H*-imidazol-2-yl)benzamide, Benzo(a)pyrene, and dorsomorphin, etc. The LRTM2 predicted 35 therapeutic drugs, including Aflatoxin B1, Benzo(a)pyrene, and bisphenol A, etc.

**Table 2:** The potential therapeutic drugs for WT were predicted by the 4 hub genes

Hub gene	Chemical name	Chemical ID	Interaction count
ADGRG2	Benzo(a)pyrene	D001564	8
ADGRG2	Aflatoxin B1	D016604	5
ADGRG2	Tetrachlorodibenzodioxin	D013749	5
ADGRG2	4-(5-Benzo(1,3)dioxol-5-yl-4-pyridin-2-yl-1*H*-imidazol-2-yl)benzamide	C459179	4
ADGRG2	Bisphenol A	C006780	4
ADGRG2	Cisplatin	D002945	4
ADGRG2	Dietary fats	D004041	4
ADGRG2	Dorsomorphin	C516138	4
ADGRG2	Phenobarbital	D010634	4
ADGRG2	Diethylnitrosamine	D004052	3
ADGRG2	Folic acid	D005492	3
ADGRG2	Valproic acid	D014635	3
ADGRG2	Choline	D002794	2
ADGRG2	Entinostat	C118739	2
ADGRG2	Furan	C039281	2
ADGRG2	Mercuric bromide	C042720	2
ADGRG2	Methapyrilene	D008701	2
ADGRG2	Methionine	D008715	2
ADGRG2	*p*-Chloromercuribenzoic acid	D020245	2
ADGRG2	Pentabromodiphenyl ether	C086401	2
ADGRG2	Phenylmercuric acetate	D010662	2
ADGRG2	Rotenone	D012402	2
ADGRG2	Tobacco smoke pollution	D014028	2
ADGRG2	1,2-Dimethylhydrazine	D019813	1
ADGRG2	2,2,5,7,8-Pentamethyl-1-hydroxychroman	C029141	1
ADGRG2	2,3′,4,4′,5-Pentachlorobiphenyl	C070055	1
ADGRG2	3,4,3′,4′-Tetrachlorobiphenyl	C028451	1
ADGRG2	3,4,5,3′,4′-Pentachlorobiphenyl	C023035	1
ADGRG2	8-Bromo cyclic adenosine monophosphate	D015124	1
ADGRG2	Abrine	C496492	1
ADGRG2	Acetamide	C030686	1
ADGRG2	Acetaminophen	D000082	1
ADGRG2	Amphetamine	D000661	1
ADGRG2	Antigens, polyomavirus transforming	D000952	1
ADGRG2	Antirheumatic agents	D018501	1
ADGRG2	Arsenic trioxide	D000077237	1
ADGRG2	Benzo(e)pyrene	C026487	1
ADGRG2	Betaine	D001622	1
ADGRG2	beta-Naphthoflavone	D019324	1
ADGRG2	Butyraldehyde	C018475	1
ADGRG2	Cadmium	D002104	1
ADGRG2	Chlorodiphenyl (54% chlorine)	D020111	1
ADGRG2	Cuprizone	D003471	1
ADGRG2	Cyclosporine	D016572	1
ADGRG2	Decitabine	D000077209	1
ADGRG2	Dexamethasone	D003907	1
ADGRG2	Doxorubicin	D004317	1
ADGRG2	Endosulfan	D004726	1
ADGRG2	Estradiol	D004958	1
ADGRG2	Fenretinide	D017313	1
ADGRG2	Formaldehyde	D005557	1
ADGRG2	Gallic acid	D005707	1
ADGRG2	Indole-3-carbinol	C016517	1
ADGRG2	Isotretinoin	D015474	1
ADGRG2	Jinfukang	C544151	1
ADGRG2	Methylmercuric chloride	C004925	1
ADGRG2	Muraglitazar	C500085	1
ADGRG2	Orphenadrine	D009966	1
ADGRG2	Oxygen	D010100	1
ADGRG2	Ozone	D010126	1
ADGRG2	Palm oil	D000073878	1
ADGRG2	Paraquat	D010269	1
ADGRG2	Particulate matter	D052638	1
ADGRG2	Perfluorooctane sulfonic acid	C076994	1
ADGRG2	Plant extracts	D010936	1
ADGRG2	Propylthiouracil	D011441	1
ADGRG2	Rosiglitazone	D000077154	1
ADGRG2	Sodium dodecyl sulfate	D012967	1
ADGRG2	Sodium fluoride	D012969	1
ADGRG2	Sunitinib	D000077210	1
ADGRG2	Systhane	C446685	1
ADGRG2	Tamoxifen	D013629	1
ADGRG2	Tesaglitazar	C501413	1
ADGRG2	Testosterone propionate	D043343	1
ADGRG2	Thioacetamide	D013853	1
ADGRG2	Triclosan	D014260	1
ADGRG2	Trimellitic anhydride	C015559	1
ADGRG2	Triphenyl phosphate	C005445	1
ADGRG2	Triptonide	C084079	1
ADGRG2	Vinylidene chloride	C029297	1
ADGRG2	Vitamin B12	D014805	1
ADGRG2	Zinc	D015032	1
CPD	Bisphenol A	C006780	6
CPD	Testosterone	D013739	5
CPD	Tetrachlorodibenzodioxin	D013749	5
CPD	Gasoline	D005742	4
CPD	Particulate matter	D052638	4
CPD	Polycyclic aromatic hydrocarbons	D011084	4
CPD	Ethanol	D000431	3
CPD	Ethinyl estradiol	D004997	3
CPD	Nitric oxide	D009569	3
CPD	Tobacco smoke pollution	D014028	3
CPD	Valproic acid	D014635	3
CPD	Abrine	C496492	2
CPD	Aflatoxin B1	D016604	2
CPD	Benzo(a)pyrene	D001564	2
CPD	Estradiol	D004958	2
CPD	Ethylene dichloride	C024565	2
CPD	Fluorouracil	D005472	2
CPD	Folic acid	D005492	2
CPD	Gardiquimod	C546771	2
CPD	Lipopolysaccharide, *E. coli* O55-B5	C482199	2
CPD	Magnetite nanoparticles	D058185	2
CPD	Pirinixic acid	C006253	2
CPD	Succimer	D004113	2
CPD	Trichostatin A	C012589	2
CPD	1,1,1-Trichloroethane	C024566	1
CPD	1,2-Dimethylhydrazine	D019813	1
CPD	1-Butanol	D020001	1
CPD	2,3,5-(Triglutathion-*S*-yl)hydroquinone	C078765	1
CPD	4-(5-Benzo(1,3)dioxol-5-yl-4-pyridin-2-yl-1*H*-imidazol-2-yl)benzamide	C459179	1
CPD	Ammonium chloride	D000643	1
CPD	Bilirubin	D001663	1
CPD	Butyraldehyde	C018475	1
CPD	Cacodylic acid	D002101	1
CPD	Calcitriol	D002117	1
CPD	Cannabidiol	D002185	1
CPD	Carbon tetrachloride	D002251	1
CPD	Chlorine	D002713	1
CPD	Chloropicrin	C100187	1
CPD	Chloroprene	D002737	1
CPD	Choline	D002794	1
CPD	Clorgyline	D003010	1
CPD	Copper	D003300	1
CPD	Cyclosporine	D016572	1
CPD	Dibutyl phthalate	D003993	1
CPD	Dietary fats	D004041	1
CPD	Dorsomorphin	C516138	1
CPD	Doxorubicin	D004317	1
CPD	Endosulfan	D004726	1
CPD	Gentamicins	D005839	1
CPD	Hexabromocyclododecane	C089796	1
CPD	Hexachlorocyclohexane	D001556	1
CPD	Hydrogen cyanide	D006856	1
CPD	Ionomycin	D015759	1
CPD	Isobutyl alcohol	C040507	1
CPD	Isotretinoin	D015474	1
CPD	Ivermectin	D007559	1
CPD	Jinfukang	C544151	1
CPD	Lactic acid	D019344	1
CPD	Methapyrilene	D008701	1
CPD	Methionine	D008715	1
CPD	Methylmercuric chloride	C004925	1
CPD	*N*-(2-(1,1′-Bicyclopropyl)-2-ylphenyl)-3-(difluoromethyl)-1-methyl-1*H*-pyrazole-4-carboxamide	C583365	1
CPD	*N*-Methyl-3,4-methylenedioxyamphetamine	D018817	1
CPD	Ozone	D010126	1
CPD	Perfluorooctanoic acid	C023036	1
CPD	Phenobarbital	D010634	1
CPD	Plant extracts	D010936	1
CPD	Potassium cyanide	D011190	1
CPD	Procymidone	C035988	1
CPD	Propylthiouracil	D011441	1
CPD	Protein kinase inhibitors	D047428	1
CPD	SandostatinLAR	C541923	1
CPD	Selenium	D012643	1
CPD	Silicon dioxide	D012822	1
CPD	Sodium arsenate	C009277	1
CPD	Sulforaphane	C016766	1
CPD	T-2 toxin	D013605	1
CPD	Tetrachloroethylene	D013750	1
CPD	Tetradecanoylphorbol acetate	D013755	1
CPD	Tretinoin	D014212	1
CPD	Trichloroethylene	D014241	1
CPD	Triptonide	C084079	1
CPD	Troglitazone	D000077288	1
CPD	Tungsten	D014414	1
CPD	Tunicamycin	D014415	1
CPD	Urethane	D014520	1
CPD	Vehicle emissions	D001335	1
CPD	Venlafaxine hydrochloride	D000069470	1
CPD	Vinclozolin	C025643	1
CPD	Vinylidene chloride	C029297	1
CPD	Zoledronic acid	D000077211	1
CTHRC1	Valproic acid	D014635	12
CTHRC1	4-(5-Benzo(1,3)dioxol-5-yl-4-pyridin-2-yl-1*H*-imidazol-2-yl)benzamide	C459179	7
CTHRC1	Benzo(a)pyrene	D001564	7
CTHRC1	Dorsomorphin	C516138	7
CTHRC1	Tetrachlorodibenzodioxin	D013749	6
CTHRC1	Cyclosporine	D016572	4
CTHRC1	Aflatoxin B1	D016604	3
CTHRC1	Bisphenol A	C006780	3
CTHRC1	(+)-JQ1 compound	C561695	3
CTHRC1	Trichostatin A	C012589	3
CTHRC1	Vorinostat	D000077337	3
CTHRC1	Belinostat	C487081	2
CTHRC1	Decitabine	D000077209	2
CTHRC1	Entinostat	C118739	2
CTHRC1	Furan	C039281	2
CTHRC1	Mercuric bromide	C042720	2
CTHRC1	Oxaliplatin	D000077150	2
CTHRC1	Phenylmercuric acetate	D010662	2
CTHRC1	Tobacco smoke pollution	D014028	2
CTHRC1	Topotecan	D019772	2
CTHRC1	Tretinoin	D014212	2
CTHRC1	1-Naphthylisothiocyanate	D015058	1
CTHRC1	2′,3,3′,4′,5-Pentachloro-4-hydroxybiphenyl	C111118	1
CTHRC1	2,3-Pentanedione	C013186	1
CTHRC1	4-(4-((5-(4,5-Dimethyl-2-nitrophenyl)-2-furanyl)methylene)-4,5-dihydro-3-methyl-5-oxo-1*H*-pyrazol-1-yl)benzoic acid	C584509	1
CTHRC1	Air pollutants	D000393	1
CTHRC1	Aluminum	D000535	1
CTHRC1	Ammonium chloride	D000643	1
CTHRC1	Antirheumatic agents	D018501	1
CTHRC1	Aristolochic acid I	C000228	1
CTHRC1	Arsenite	C015001	1
CTHRC1	Atrazine	D001280	1
CTHRC1	bis(4-Hydroxyphenyl)sulfone	C543008	1
CTHRC1	Bisphenol F	C000611646	1
CTHRC1	Butyraldehyde	C018475	1
CTHRC1	Calcitriol	D002117	1
CTHRC1	Calcium chloride	D002122	1
CTHRC1	Carbon tetrachloride	D002251	1
CTHRC1	Chloroprene	D002737	1
CTHRC1	Cisplatin	D002945	1
CTHRC1	Copper sulfate	D019327	1
CTHRC1	Deoxynivalenol	C007262	1
CTHRC1	Dicrotophos	C000944	1
CTHRC1	Diethylhexyl phthalate	D004051	1
CTHRC1	Diethylnitrosamine	D004052	1
CTHRC1	Doxorubicin	D004317	1
CTHRC1	Estradiol	D004958	1
CTHRC1	Ethanol	D000431	1
CTHRC1	Ethinyl estradiol	D004997	1
CTHRC1	Ethylene dichloride	C024565	1
CTHRC1	Folic acid	D005492	1
CTHRC1	Gemcitabine	C056507	1
CTHRC1	Genistein	D019833	1
CTHRC1	Glycidol	C004312	1
CTHRC1	Hexabromocyclododecane	C089796	1
CTHRC1	ICG 001	C492448	1
CTHRC1	Ionomycin	D015759	1
CTHRC1	Isoproterenol	D007545	1
CTHRC1	Milrinone	D020105	1
CTHRC1	Monomethyl phthalate	C517284	1
CTHRC1	MRK 003	C523799	1
CTHRC1	Naphthalene	C031721	1
CTHRC1	*n*-Butoxyethanol	C017096	1
CTHRC1	*n*-Hexane	C026385	1
CTHRC1	Panobinostat	D000077767	1
CTHRC1	Paraquat	D010269	1
CTHRC1	Particulate matter	D052638	1
CTHRC1	Pirinixic acid	C006253	1
CTHRC1	Propionaldehyde	C005556	1
CTHRC1	Quercetin	D011794	1
CTHRC1	Resveratrol	D000077185	1
CTHRC1	*S*-2-Pentyl-4-pentynoic hydroxamic acid	C513635	1
CTHRC1	Silver	D012834	1
CTHRC1	Smoke	D012906	1
CTHRC1	Testosterone	D013739	1
CTHRC1	Tetradecanoylphorbol acetate	D013755	1
CTHRC1	Titanium dioxide	C009495	1
CTHRC1	Trichloroethylene	D014241	1
CTHRC1	Trimellitic anhydride	C015559	1
CTHRC1	tris(2-Butoxyethyl) phosphate	C013320	1
CTHRC1	Tungsten compounds	D017973	1
LRTM2	Aflatoxin B1	D016604	5
LRTM2	Benzo(a)pyrene	D001564	4
LRTM2	Bisphenol A	C006780	3
LRTM2	Benz(a)anthracene	C030935	2
LRTM2	Carbon tetrachloride	D002251	2
LRTM2	Dietary fats	D004041	2
LRTM2	Nanotubes, carbon	D037742	2
LRTM2	Tetrachlorodibenzodioxin	D013749	2
LRTM2	2,2,5,7,8-Pentamethyl-1-hydroxychroman	C029141	1
LRTM2	Benzo(b)fluoranthene	C006703	1
LRTM2	Benzo(e)pyrene	C026487	1
LRTM2	Cadmium chloride	D019256	1
LRTM2	Choline	D002794	1
LRTM2	Chrysene	C031180	1
LRTM2	Diethyl maleate	C014476	1
LRTM2	Doxorubicin	D004317	1
LRTM2	Endosulfan	D004726	1
LRTM2	Ethylene dichloride	C024565	1
LRTM2	Folic acid	D005492	1
LRTM2	Furan	C039281	1
LRTM2	Methapyrilene	D008701	1
LRTM2	Methionine	D008715	1
LRTM2	Palm oil	D000073878	1
LRTM2	Rotenone	D012402	1
LRTM2	Sodium bichromate	C016104	1
LRTM2	Systhane	C446685	1
LRTM2	Testosterone	D013739	1
LRTM2	Thioacetamide	D013853	1
LRTM2	Tobacco smoke pollution	D014028	1
LRTM2	Trichloroethylene	D014241	1
LRTM2	Triptonide	C084079	1
LRTM2	tris(1,3-Dichloro-2-propyl)phosphate	C016805	1
LRTM2	Valproic acid	D014635	1
LRTM2	Vehicle emissions	D001335	1
LRTM2	Zinc oxide	D015034	1

### Validation of the gene expression level by qRT-PCR

**Fig. 9:**
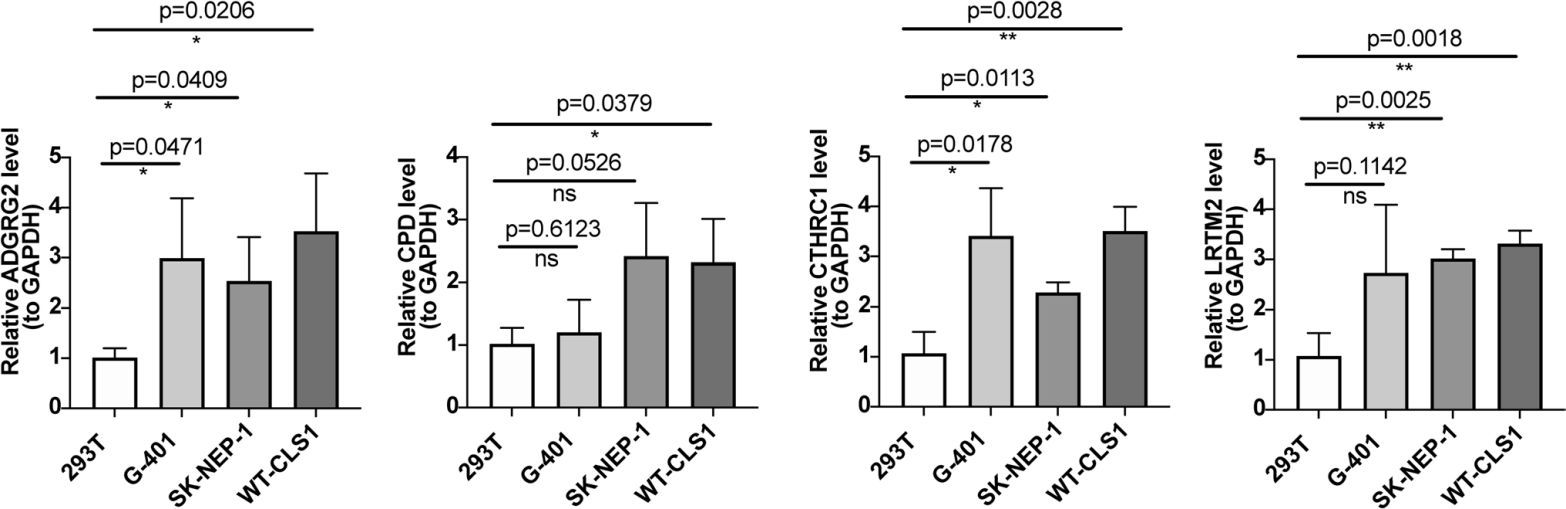
QRT-PCR experiments to verify the expression levels of four hub genes in the four cell lines. The statistic analysis was performed by Unpaired t-test, and the data was exhibited as mean SD (*P* < 0.05)

To further verify the expression level of the 4 hub genes (ADGRG2, CPD, CTHRC1, and LRTM2), we used qRT-PCR to compare gene expression levels in the four cell lines. The qRT-PCR results showed that the expression level of four genes in the 293T cell line was all lower, while the expression level of ADGRG2 was higher in the WT-CLS1 cell line, and the expression levels of CTHRC1 were higher in the G-401 cell line and WT-CLS1 cell line (Fig. 9).

## Discussion

The pathogenesis of WT is still unclear, and the related epigenetic mechanism remains to be explored. As a post-transcriptional gene expression regulation model, m6A RNA methylation regulates gene expression by affecting multiple aspects of mRNA metabolism, including mRNA pre-processing, nuclear export, decay, and translation [28]. Therefore, the effect of m6A on gene expression is extensive. In other tumor studies, it was found that m6A in the peripheral blood of patients with gastric cancer increased with the progression and metastasis of gastric cancer but decreased significantly after surgery, suggesting that m6A level in peripheral blood is a promising noninvasive diagnostic biomarker for gastric cancer patients [29]. In the study of m6A RNA methylation in glioma, it was found that high expression of m6A was associated with poor prognosis and tumor grade [30]. In the study of urological malignancies, high levels of m6A RNA methylation in mRNA were found to promote prostate cancer progression by regulating subcellular protein localization, and patients with high m6A RNA methylation had poor survival benefits [31]. Our study found that m6A is up-regulated in nephroblastoma, and increased expression of m6A is associated with poor prognosis and is associated with the grade of nephroblastoma because the expression of m6A in grade III and IV is more robust than that in grade I and II. These findings suggest that abnormal methylation of m6A may show specific diagnostic biomarkers and prognostic value in nephroblastoma. In addition, m6A is reversible, and reversing high m6A expression may contribute to cancer treatment [32]. However, the fundamental question of the factors causing m6A deposition remains unanswered [33], suggesting that the observed overexpression of m6A RNA methylation in WT patients requires further investigation.

The dysregulation or mutation of m6A “writer” and “eraser” proteins is closely associated with m6A deposition in cancer cells. According to reports, the m6A “writer” proteins methyltransferase-like 3 (METTL3) and METTL14, as well as the “eraser” protein obesity-associated protein (FTO), are intricately involved in the modulation of m6A methylation and the development of various cancer types [34]. Dysregulated transcriptional regulation in cancer cells can also impact m6A deposition. Transcriptional regulators associated with cancer can potentially enhance the expression of m6A “writer” proteins, consequently leading to elevated m6A levels [35]. Furthermore, accumulating evidence indicates that various environmental factors can influence m6A deposition. For example, it has been demonstrated that UV irradiation can induce the formation of m6A methylation [33]. However, the fundamental question regarding the underlying factors leading to m6A deposition remains inadequately addressed, lacking a comprehensive and definitive elucidation [33], suggesting that the intricate mechanisms underlying the interplay of these transcriptional, mutational, and environmental factors with altered m6A RNA methylation in WT patients requires further investigation.

In recent years, more and more studies have confirmed that m6A-related genes are closely related to malignant tumors. For example, ZC3H13, CBLL1, ELAVL1, and YTHDF1 are differentially expressed in lung cancer tissues, which is significant for predicting and treating lung cancer [36]. Nine m6A-related genes were identified in glioma. The mRNA levels of these genes were highly correlated with the clinicopathological features of glioma and may be involved in glioma progression [37]. In the head and neck squamous cell carcinoma study, m6A-related gene changes were significantly correlated with tumor grade and stage [38]. Although the abnormal expression of m6A-related genes is involved in tumorigenesis in many solid tumors, the prognostic value of m6A-related genes in nephroblastoma and its correlation with clinicopathological features still need further study. We screened four highly expressed m6A-related genes (ADGRG2, CPD, CTHRC1, LRTM2) in nephroblastoma and constructed an effective diagnostic model based on these genes. In addition, we used qRT-PCR to verify the expression of m6A-related genes in different WT cell lines and normal 293T cell lines. The results showed that the expression of these four genes in normal 293T cell lines was lower than in different WT cell lines. It is suggested that these four m6A-related genes can be used as biomarkers for diagnosing WT and potential therapeutic targets. Although our study focused on m6A-related genes, we found in many results of our investigations that the DEGs between WT tissues and normal tissues partially overlapped with another study. A study by Li et al. compared the DEGs between WT and normal tissues from the GEO (Gene Expression Omnibus) database by bioinformatics methods, and 10 DEGs (ALB, CDH1, EGF, AQP2, REN, SLC2A2, SPP1, UMOD, NPHS2, and FOXM1) were screened [39]. The AQP2, SPP1, and UMOD in the results of this study coincided with the DEGs we screened. Differences in AQP2, SPP1, and UMOD between WT and normal tissues were found in two different databases, and our study provides additional evidence to support AQP2, SPP1, and UMOD as DEGs of WT.

We reviewed the research status of these four highly expressed m6A-related genes. The correlation between CTHRC1 and tumor is the most studied, followed by ADGRG2, while the correlation between CPD and LRTM2 and tumor is less studied. CTHRC1 is a vital oncogene; its expression of collagen triple helix repeat containing protein 1 is a cancer-related protein. CTHRC1 is the most widely studied and has been consistently shown to be upregulated across multiple cancer types (e.g., gastric cancer, pancreatic cancer, hepatocellular carcinoma, breast cancer, colorectal cancer, epithelial ovarian cancer, esophageal squamous cell carcinoma, cervical cancer, non-small cell lung cancer, melanoma), aligning with our observation of its elevated expression in WT [40–43]. Increased CTHRC1 expression has consistently been associated with tumor development, and its expression level significantly correlates with the prognosis of cancer patients [41]. Qi et al. first confirmed the high expression of CTHRC1 in WT tumors, and further studies have found that the survival time of patients with increased expression of CTHRC1 is shorter than that of patients with low expression of CTHRC1, and CTHRC1 can be regarded as an independent prognostic factor for WT [42]. However, a discrepancy is noted in the findings by Huang et al. that despite the high expression of CTHRC1 in WT compared to normal kidney tissue, it acts as a protective factor (HR = 0.489) associated with WT prognosis [43]. This difference could be attributed to patient cohort characteristics, highlighting the need for further validation. Our study also confirmed the high expression of CTHRC1 in WT and found that CTHRC1 significantly differed in different stages of WT. The expression of CTHRC1 in grades I and II was higher than in grades III and IV. Next, we performed a survival analysis of CTHRC1, and the results showed that patients with increased expression of CTHRC1 in WT patients had a better prognosis than patients with low expression. These results suggest that the difference in CTHRC1 expression may be related to the prognosis of WT patients. CTHRC1 is a protective or risk factor for WT; further research is needed.

ADGRG2 is considered a new pathogenic gene, and most studies have shown that it is closely related to the congenital absence of vas deferens, and there are currently no studies on WT [44, 45]. The research on CPD is limited to plant research, and there is no research involving CPD and WT [46]. Furthermore, studies investigating the correlation between LRTM2 and tumors are scarce, necessitating further research to elucidate its function in this context. Overall, this study reinforces prior knowledge of dysregulated m6A-gene expression in malignancies and reveals new WT-specific prognostic biomarkers that contribute uniquely to the current understanding of m6A biology in pediatric cancer. While our analysis was focused on m6A-related genes, the identified DEGs between tumor and normal samples also provide valuable insights. The abnormal m6A modifications can directly impact target RNA structure and function, potentially contributing to the altered gene expression patterns observed.

Conversely, the proteins encoded by DEGs may have feedback to affect the m6A process. Exploring the interactions between m6A dysregulation and downstream DEG targets could shed light on the collective molecular changes driving nephroblastoma pathogenesis. Connecting the DEGs-enriched pathways to the upstream effects of m6A on modulating gene expression represents an important future direction to dissect the complex interplay underlying Wilms tumor biology.

Understanding the immune status of the tumor microenvironment will help us deepen the understanding of anti-tumor immune responses and develop more effective immunotherapy methods. This study found that APC_co_stimulation, CCR, Macrophages, Parainflammation, Th1_cells, Treg, and Type_ II_IFN_response were significantly decreased in WT compared with normal tissues. This suggests that overall immunosuppression in the WT tumor microenvironment is consistent with many current findings [47]. Some studies have compared the differences of immune cells in tumor microenvironment between the WT high-risk and low-risk groups. In the high-risk group, it was found that except for B cells and macrophage M1 type, other types of cells were lower than those in the low-risk group, including CD8+ naive T cells, CD8+ T cells, macrophage M2 type, mast cells, neutrophils, NKT cells and Treg cells [48]. This suggests that the high-risk WT tumor microenvironment has immune inactivation and a lack of T cells.

Furthermore, our study unraveled significant correlations between specific immune markers and the m6A-related genes. ADGRG2, as a G protein-coupled receptor, may be correlated with immune infiltrating cells, and its overexpression in tumors is generally associated with lower overall survival rates [49]. CTHRC1 is overexpressed in multiple tumor types to promote tumor initiation and progression. It regulates the activity of immune checkpoint genes through various signaling pathways and is associated with immune cell infiltration and the tumor microenvironment [41]. There is limited research on CPD and LRTM2 in the context of tumors. Still, it has been reported that CPD cleavage of C-terminal arginine generates nitric oxide, which plays a versatile regulatory role in various physiological processes and is closely associated with tumor invasion and tumor-induced angiogenesis [50]. In addition, we found that APC_co_stimulation, CCR, Macrophages, Parainflammation, Treg, and Type_II_IFN_Reponse were negatively correlated with LRTM2, Th1_cells were positively correlated with ADGRG2, CCR was negatively correlated with CPD, CCR was positively correlated with CTHRC1. These findings indicate that the four m6A-related genes could play an immunoregulation role in WT. In particular, LRTM2 may be involved in the immunosuppressive microenvironment of WT. Although the fact that there are few studies on LRTM2 in WT, it has potential research value in the future.

While this study provides valuable initial insights into m6A-associated prognostic biomarkers in Wilms tumor, there are some limitations regarding the study design. The analysis was performed on a single cohort of samples from the TARGET-WT database. We could not validate our findings on an independent GEO dataset due to the lack of publicly available cohorts with both Wilms tumor gene expression and survival outcome data. As more annotated datasets become available in resources like GEO, validation on external samples will be an important future direction to substantiate the prognostic utility of the identified biomarkers. Furthermore, the sample size of 121 WT patients, while sizable given the rarity of this pediatric cancer, may still limit the detection of more minor effects. Integrating multi-omic data beyond just transcriptomics could provide a more comprehensive understanding of the functional mechanisms of the identified biomarkers. Overall, this exploratory study offers a meaningful starting point for further research to build upon and more definitively characterize the clinical and biological significance of m6A-related genes in Wilms tumor.

Enhancing the immune activity of the WT tumor microenvironment may contribute to the treatment of WT. Still, it should be noted that different target cells have various therapeutic effects on WT. Immune checkpoint inhibitors (PD1 or PD-L1) have completely changed the treatment of many adult tumors because they can activate tumor-infiltrating lymphocytes to exert anti-tumor effects. People have placed similar hopes on treating recurrent or refractory solid tumors in children. However, current clinical trials have shown that this immunotherapy is little effective in treating WT, and childhood cancers are likely to follow a unique immune pathway [51]. This means that immune checkpoint inhibitors may not be suitable for treating WT. Other immunotherapy methods for WT are being explored, such as CAR-T cell therapy and cytotoxic T lymphocyte therapy. This immunotherapy for T cells shows a specific prospect and may be successfully applied to WT immunotherapy in the future [52]. Previous studies have primarily focused on applying immune checkpoint inhibitors in Wilms tumor (WT), while our research provides a novel perspective on WT immunotherapy. Our findings demonstrate a strong association between m6A-related genes and the immunosuppressive state of the WT tumor microenvironment, providing a theoretical foundation for developing immunotherapeutic strategies targeting these genes. Notably, the LRTM2 gene may become a promising target as it correlates negatively with multiple immune gene sets. Modulating LRTM2 could potentially enhance immune activity within the WT microenvironment.

Additionally, we predicted the potential therapeutic effects of these key genes, providing valuable leads for developing immunotherapeutic drugs for WT. In summary, this study comprehensively elucidates the WT immune microenvironment, uncovering the challenges faced by WT immunotherapy and offering essential data to support the development of novel treatment targets and drugs. It represents an innovative contribution to the field of WT immunotherapy.

## Conclusion

In summary, four m6A-related genes were screened out by bioinformatics analysis of RNA-seq data of WT patients. After experimental verification, we found that ADGRG2, CPD, CTHRC1, and LRTM2 were highly expressed in WT and could have potential prognostic value and play an immunoregulation role in WT. These findings help to deepen the understanding of the molecular mechanism of WT and provide potential targets for clinical treatment in the future.

## Data Availability

The datasets generated during and/or analyzed during the current study are available from the corresponding author upon reasonable request.
